# Apport et limites de l’intelligence artificielle dans la rédaction d’articles scientifiques : attention à l’imposture

**DOI:** 10.48327/mtsi.v6i2.2026.843

**Published:** 2026-04-15

**Authors:** Jean-Philippe CHIPPAUX

**Affiliations:** IRD – MERIT, Université Paris Cité, F-75006 Paris, France

**Figure:**
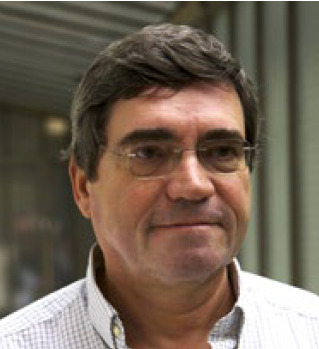

Depuis quelques mois, la rédaction de *Médecine Tropicale et Santé Internationale* (*MTSI*), comme celle de nombreuses autres revues scientifiques, est confrontée à l’afflux de manuscrits dont la préparation ou la rédaction ont manifestement eu recours à l’intelligence artificielle (IA). Pour autant, rares sont les auteurs qui le mentionnent. Ce silence dénote un manque d’éthique et d’intégrité scientifique d’autant plus préjudiciable que la plupart des auteurs en méconnaissent les limites. Son utilisation inconsidérée les expose à des erreurs générées par l’IA elle-même, entraînant un rejet du manuscrit [2,4]. Il convient de rappeler que les auteurs sont solidairement responsables de leur travail et de leur manuscrit.

L’IA peut s’avérer performante dans la synthèse de documents, la recherche de références, l’organisation d’un argumentaire, l’amélioration du style et la correction de fautes de grammaire ou d’orthographe [3]. La rédaction de MTSI n’est pas opposée à son utilisation. Elle demande simplement à ce qu’elle soit déclarée et ses modalités précisées au moment de la soumission du manuscrit conformément aux recommandations aux auteurs [1]. Il faut, cependant, bien connaître le fonctionnement de l’IA pour, d’une part guider sa recherche par une interrogation appropriée en amont (grâce aux « prompts ») et, d’autre part, déceler les déviations ou insuffisances de ses réponses (dont les « hallucinations »).

L’IA ne produit pas un résultat logique ou rationnel – et encore moins original – mais la solution la plus probable selon un algorithme et des critères statistiques – confidentiels – définis par le fabricant du logiciel et exempts de tout esprit critique [1]. Ses affirmations constituent une somme d’allégations non vérifiées. L’IA puise ses sources sur Internet, sans hiérarchie, ni cohérence. Elle ne vérifie pas l’authenticité des informations et met sur un même plan, les études scientifiques fondées sur une méthodologie rigoureuse dûment validée, et des informations dépourvues de preuve ou d’analyse objective.

En outre, les sources que l’IA cite comme justification peuvent avoir été créées de toutes pièces – ce que l’on appelle « hallucinations » – avec une trompeuse apparence de vraisemblance. Ces fausses références affichent des auteurs reconnus comme spécialistes du domaine, un titre parfaitement cohérent avec le sujet et le nom d’une revue scientifique dont l’année, le volume et le fascicule sont exacts. Cependant, aux pages indiquées, ce n’est pas l’article cité que l’on trouve mais un autre dont le thème est sans rapport avec celui du manuscrit. Parfois, auteurs, titre et/ou journal sont fictifs. La référence inventée ne soutient pas le propos de l’auteur. En revanche, elle révèle sa fraude et son manque d’intégrité scientifique, jetant un sérieux doute sur l’ensemble de son travail. Elle démontre que l’auteur n’a pas pris la peine de consulter les sources qu’il cite puisque certaines n’existent pas.

Pour s’en préserver, l’auteur doit définir le cadre de sa recherche et délimiter son champ d’investigation avant toute interrogation de l’IA. C’est le rôle des prompts et des documents fournis à l’IA comme sources qui doivent être choisis avec soin et pertinence. Cela requiert que l’auteur les ait préalablement identifiés, sélectionnés et analysés. Par ailleurs, si l’IA fournit des sources bibliographiques, l’auteur doit les vérifier scrupuleusement. Il s’assurera de l’existence de chacune d’elles et examinera attentivement leur contenu pour confirmer que les données et les opinions présentées éclairent son propos (Encadré).

Encadré Recommandations du Comité international des rédacteurs de revues médicales (ICMJE) concernant l'utilisation des technologies assistées par l'IA (d’après [2])

- Lorsqu'ils soumettent un manuscrit, les auteurs doivent indiquer s'ils ont utilisé des technologies assistées par l'IA. Le nom de la plateforme, sa version et le concepteur doivent être indiqués.
- Si l'IA a été utilisée pour aider à la rédaction, les auteurs doivent le mentionner dans la section « Remerciements ».
- Si l'IA a été utilisée pour la collecte de données, l'analyse ou la création de figures, les auteurs doivent le mentionner dans la section « Méthodes ».
- Les outils d'IA, y compris les chatbots tels que ChatGPT, ne doivent pas être crédités en tant qu'auteurs, car ils ne peuvent assumer la responsabilité de l'exactitude, de l'inté-grité ou de l'originalité du travail, qui sont nécessaires à la paternité de l'œuvre.
- Les auteurs doivent soigneusement réviser et modifier le contenu généré par l'IA, car ces outils peuvent produire des résultats qui semblent fiables, mais qui peuvent être faux, incomplets ou biaisés.

Face à cette menace croissante, la rédaction de *MTSI* n’a d’autre choix que de refuser systématiquement tout manuscrit ayant utilisé l’IA au cours de sa préparation, sans le déclarer et le justifier. Toute référence frauduleuse (hallucination) constatée dès la première version entraînera le rejet définitif du manuscrit sans examen supplémentaire.
