# Supplementary material for: The benefits and limitations of artificial intelligence in scientific writing: beware of fraud
Source: Med Trop Sante Int. 2026 Apr 15;6(2):mtsi.v6i2.2026.843. [Article in French] doi: 10.48327/mtsi.v6i2.2026.843 (PMC13423300; doi:10.48327/mtsi.v6i2.2026.843)
Supplement: Related texts

# Research article: The benefits and limitations of artificial intelligence in scientific writing: beware of fraud

In recent months, the editorial board of *Médecine Tropicale et Santé Internationale* (*MTSI*) has experienced a surge in manuscripts that clearly involved the use of artificial intelligence (AI) in their preparation or drafting, as have the editorial boards of many other scientific journals. However, few authors mention this. This silence indicates a lack of scientific ethics and integrity, which is particularly problematic given that most authors are unaware of AI’s limitations. Reckless use of AI exposes authors to errors generated by the technology itself, leading to manuscript rejection [2,4]. Authors are jointly responsible for their work and manuscript.

AI can effectively summarize documents, search for references, organize arguments, improve style, and correct grammar and spelling errors [3]. The MTSI editorial board is not opposed to its use. It simply requires that its use be declared and its specifics outlined at the time of manuscript submission, in accordance with the author guidelines [1].

However, one must thoroughly understand how AI works to guide its search with appropriate upstream queries (“prompts”) and detect deviations or shortcomings in its responses (“hallucinations”).

AI does not produce logical, rational, or original results. Rather, it provides the most probable solution according to confidential algorithms and statistical criteria defined by the software manufacturer. These criteria are devoid of critical thinking [1]. Its assertions are a collection of unverified claims. AI draws its sources from the internet without any hierarchy or coherence. It does not verify the authenticity of the information, treating scientific studies based on rigorous, validated methodology the same as information lacking evidence or analysis.

Furthermore, the sources that AI cites as justification may be fabricated—”hallucinations” with a deceptively plausible appearance. These false references list authors recognized as experts in the field with titles consistent with the subject and the name of a scientific journal with the correct year, volume, and issue number. However, on the indicated pages, the cited article is not found, but rather, another article whose topic is unrelated to that of the manuscript. Sometimes, the authors, title, and/or journal are fictitious. The fabricated reference does not support the author’s argument. In fact, it reveals their fraud and lack of scientific integrity, casting serious doubt on their entire body of work. This demonstrates that the author did not bother to consult the cited sources, since some do not exist.

To avoid this, authors must define the scope of their research and limit their field of investigation before querying the AI. This is the purpose of the prompts and documents that are provided to the AI as sources. These sources must be chosen carefully and be relevant. The author must have previously identified, selected, and analyzed these sources.

Furthermore, if the AI provides bibliographic sources, the author must scrupulously verify them. The author must confirm the existence of each source and carefully examine its content to ensure that the data and opinions presented support their argument (Box).

Box Recommendations regarding the use of AI-assisted technologies are adapted from the International Committee of Medical Journal Editors (ICMJE) (adapted from [2])

- When submitting a manuscript, authors must disclose whether they have used AI-assisted technologies. Specify the name of the platform, its version, and the developer.
- If AI was used for writing assistance, authors must mention this in the "Acknowledgments" section.
- If AI was used for data collection, analysis, or creating figures, authors must mention this in the "Methods" section.
- AI tools, including chatbots such as ChatGPT, should not be credited as authors because they cannot assume responsibility for the accuracy, integrity, or originality of the work, all of which are necessary for authorship.
- Authors must carefully review and edit AI-generated content because these tools can produce results that appear reliable but may be false, incomplete, or biased.

In light of this growing threat, the *MTSI* editorial board must systematically reject any manuscript that uses AI during preparation without declaring and justifying its use. Any fraudulent reference (hallucination) found in the first draft will result in definitive rejection of the manuscript without further review.
